# Artificial Intelligence in Cutaneous Oncology

**DOI:** 10.3389/fmed.2020.00318

**Published:** 2020-07-10

**Authors:** Yu Seong Chu, Hong Gi An, Byung Ho Oh, Sejung Yang

**Affiliations:** ^1^Department of Biomedical Engineering, Yonsei University, Wonju, South Korea; ^2^Department of Dermatology and Cutaneous Biology Research Institute, Yonsei University College of Medicine, Seoul, South Korea

**Keywords:** artificial intellegence, cutaneous oncology, skin cancer, machine learning, deep learning

## Abstract

Skin cancer, previously known to be a common disease in Western countries, is becoming more common in Asian countries. Skin cancer differs from other carcinomas in that it is visible to our eyes. Although skin biopsy is essential for the diagnosis of skin cancer, decisions regarding whether or not to conduct a biopsy are made by an experienced dermatologist. From this perspective, it is easy to obtain and store photos using a smartphone, and artificial intelligence technologies developed to analyze these photos can represent a useful tool to complement the dermatologist's knowledge. In addition, the universal use of dermoscopy, which allows for non-invasive inspection of the upper dermal level of skin lesions with a usual 10-fold magnification, adds to the image storage and analysis techniques, foreshadowing breakthroughs in skin cancer diagnosis. Current problems include the inaccuracy of the available technology and resulting legal liabilities. This paper presents a comprehensive review of the clinical applications of artificial intelligence and a discussion on how it can be implemented in the field of cutaneous oncology.

## Introduction

The increasing incidence of skin cancer is a global trend. Skin cancer, which was previously known to be a common disease in Western countries, is occurring more frequently in South Korea. According to the Korean Statistical Information Service^1^, the number of patients with non-melanoma skin cancer in 2015 was 4,804 (9.4 people per 100,000), an increase over the 1,960 in 2005 and 3,270 in 2010. The increase in incidence rate is thought to be due to the aging population, the increased popularity of outdoor activities, increased ultraviolet exposure, improved access to medical services, and increased awareness of skin cancer among patients (1).

Skin biopsy and histopathologic evaluation are essential in confirming skin cancer. However, it is impossible to confirm all pigmented lesions by biopsy due to pain and scar development. Therefore, it is first necessary to establish whether or not a biopsy is required through a visual inspection performed by an experienced dermatologist. Furthermore, dermatologist needs a device that can detect changes over time in skin lesions and record the lesions in detail so that wrong-site surgery does not occur (2, 3).

With the development of imaging technologies, methods and devices for recording and analyzing what doctors see have progressed rapidly. Universally, dermoscopic imaging irradiates light onto the upper dermal layer, to observe and record more detailed pigment changes. In recent years, development of high-resolution non-invasive diagnostic devices (e.g., confocal microscopy, multiphoton microscopy, etc.) that can detect cellular levels of the skin lesions without biopsy has also been enriched (4–6). In addition, diagnoses of such skin images using artificial intelligence (AI) have been shown to outperform the average diagnosis performances of doctors. These developments are expected to have a significant impact on the diagnosis of skin cancer, the accurate recording of changes in suspicious lesions, and the effectiveness of follow-up skin cancer surgery. For user convenience, applications suitable for general smartphones have become available; however, these are not sufficiently supported by scientific evidence.

In this review, we introduce the basic concepts and clinical applications of AI via a literature review and discuss how these can be implemented in the field of dermatological oncology.

## Basic Concepts of Artificial Intelligence

AI is a field of computer science that solves problems by imitating human intelligence, these problems typically require the recognition of patterns in various data. Conventional machine learning refers to machine learning methods that do not involve deep learning; these methods extract features such as those relating to colors, textures, and edges. In conventional machine learning, precise engineering knowledge and extensive experience are required to design feature extractors capable of extracting suitable features. Using these features, conventional machine learning can derive various results and identify correlations.

Deep learning uses deep neural networks to learn features, which are obtained by designing simple but non-linear modules for each layer. Using deep neural networks, very complex functions can be learned. For example, in the field of computer vision, a deep neural network's first layer typically learns the presence of edges at particular orientations and locations within the image. Larger combinations of such edges are identified in the next layer. As the layers become deeper, they learn larger and more specific features (7).

**Figure 1:**
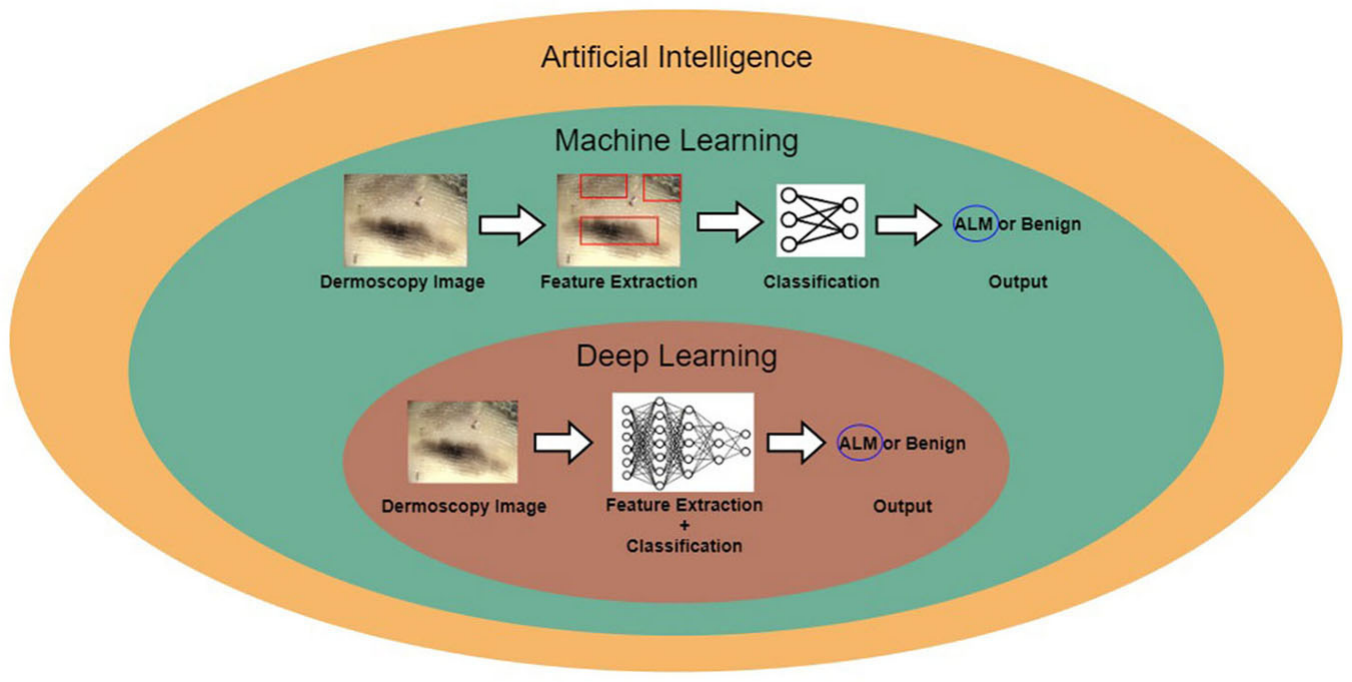
Relationship between artificial intelligence, machine learning, and deep learning.

Figure 1 shows the relationship between AI, machine learning, and deep learning. Deep learning falls within the category of machine learning, which falls within the category of AI. In this figure, the examples for conventional machine learning and deep learning are classifications of acral lentiginous melanoma (ALM) and benign nevus (BN) in dermoscopy images. Conventional machine learning extracts specific features from dermoscopy images; for example, the gray-level co-occurrence matrix (GLCM) is used to extract texture features (8). The conventional machine learning method then trains classifiers, using the extracted features to classify ALM and BN. However, deep learning methods learn by extracting various features through deep neural networks. The main difference between conventional machine learning and deep learning is that deep learning extracts various features per layer, without human intervention (9).

**Figure 2:**
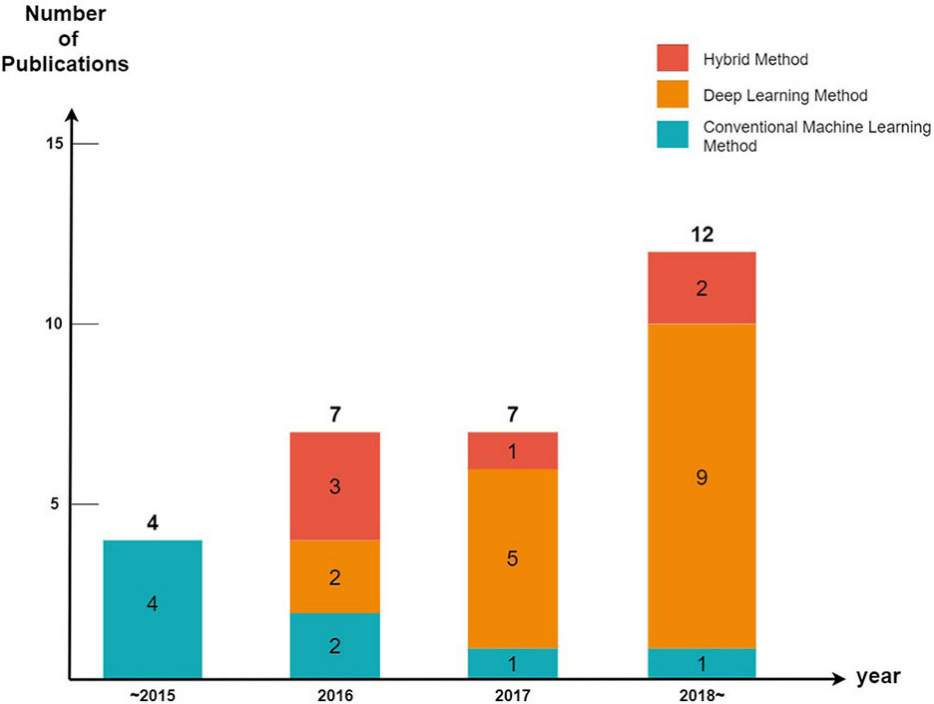
Number of publications employing artificial intelligence in cutaneous oncology.

We divided the cutaneous oncology publications into those evaluating malignant skin cancers and non-melanoma skin cancers. In addition, each publication was divided into machine learning (excluding deep learning), deep learning, and hybrid methods (a combination of machine learning and deep learning) (Figure 2).

**Figure 3:**
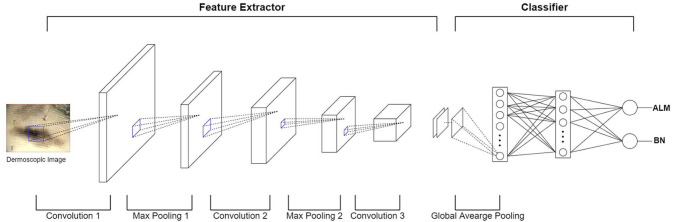
Example of DCNN for classifying ALM and BN in dermoscopic images. In the feature extractor, each layer performs a convolution operation on the input data and then performs a max-pooling operation, thereby reducing the image size and increasing the number of channels. The feature extractor generates a feature map by repeating this process for each layer. After the global average pooling operation, the feature map is used as the input of the classifier layer (fully-connected layer). Finally, the output of the fully-connected layer appears as a probability of ALM or BN.

In terms of machine learning methods, most publications use a feature extractor to extract a feature from an image, they then train the classifier model using these features (e.g., malignant melanoma (MM) vs. BN). Recently, deep convolution neural network (DCNN) have been implemented in many medical-imaging studies (10–12). DCNN use convolution operations to compensate for the problems that arise through neglecting the correlations and pixel localities of multi-layer perceptron (MLP). Thus, deep learning can be used to train a robust classifier model with a variety of data. Figure 3 shows an example of a DCNN for classifying ALMs and BNs in dermoscopic images. The DCNN feature extractor repeatedly applies convolution and max-pooling (to obtain the largest activation for each region) operations to the layer input. This process generates a feature map. The feature map is inputted to a classifier via global average pooling for each channel. The classifier finally determines probabilities for ALM and BN. The result is then compared with the actual label, and the parameters are updated via backpropagation. However, DCNN operations require highly powerful graphics processing units to manage the complex computations and large datasets involved. Although DCNN learning capacities can be limited by insufficient medical-image data, it is possible to fine-tune state-of-art deep learning models that show high performance in ImageNet large-scale visual recognition challenge (ILSVRC), making them suitable for medical purposes (13). In the hybrid method, an ensemble classifier is designed by combining a conventional machine learning method and a deep learning method. For example, after extracting the features of an image using a conventional machine learning method, these extracted features are used as inputs for a DCNN. Another example is that of training a support vector machine (SVM) using a feature map obtained through a DCNN (14). One publication showed that hybrid models outperform both deep learning and conventional machine learning models (15), another publication highlighted the limitations of deep learning and stated a need for hybrid models to overcome these limitations (16). Thus, these two methods can be used effectively to create more accurate models.

Every year, the number of articles describing AI implementations in the field of cutaneous oncology increases. By observing the trends of the discipline, it can be seen that studies using conventional machine learning have been decreasing in popularity since 2015 (five publications in 2015, three publications from 2016 to 2017, and one publication after 2018); however, the number of studies conducted using deep learning methods has increased significantly since 2015 (zero publications in 2015, seven publications from 2016 to 2017, and nine publications after 2018). These tendencies are a result of the increasing availability of big data and powerful GPUs. Since 2015, state-of-art deep learning models such as ResNet have also been studied [ResNet competed for the first time in the 2015 ILSVRC (17); it surpassed the human error rate of 5%, achieving an error rate of 3.6%].

## Application of Artificial Intelligence in the Diagnosis of Malignant Skin Cancers

### Melanoma

A total of 18 publications were identified, six of these described the use of conventional machine learning, nine publications showed the use of deep learning, and two publications presented the use of hybrid models. Among the total 18 publications, 14 used dermoscopic images as the dataset, and the remainder used unspecified or clinical images; nine used more than 500 datasets, and the remainder used <500 datasets. Moreover, in five of the publications, other skin lesion data such as seborrheic keratosis (SK) and basal cell carcinoma (BCC) were used alongside malignant melanomas and nevus. Seven publications presented the area under the curve (AUC) as a performance indicator of the model and the remainder presented accuracy (Acc), sensitivity (Sen), and specificity (Spe) (Tables 1–3).

**Table 1:** Melanoma skin cancer publications using deep learning method.

**Publication**	**End-point**	**Results**	**Method**	**Dataset**
Li et al. (18)	– Lesion segmentation (task1) – Lesion dermoscopic feature extraction (task2) – Lesion classification (task3)	Task 1: JA : 0.710 (LIN) Task 2 :AUC : 0.848 (LFN) Task 3 :AUC : 0.912 (LIN with LICU)	– Task 1 and Task 3 Preprocessing: Center crop + Resize(320*320) Data augmentation Task 1 used LIN Task 3 used LIN with LICU– Task 2 Pre-processing: Superpixel extraction Data augmentation Random sample Patch rotation Using LFN	ISIC 2017 dataset (*n* = 2000) The dataset contains melanoma, SK and nevus
Pour et al. (19)	– Lesion segmentation – Lesion dermoscopic feature segmentation (streak and globule features)	Lesion segmentation: Sen : 0.91 Spe : 0.95 Acc : 0.94 JA : 0.83 DI : 0.89 Lesion dermoscopic feature segmentation: Sen: 0.119 Spe: 0.997 Acc: 0.991 JA: 0.60 DI: 0.108	Data Augmentation – Lesion segmentation : Deeper model with 16 conv. layers, augmentation by flipping and cropping (7200 training images) – Lesion dermoscopic feature segmentation: Similar convolutional layers initialized with a pre-trained model from lesion segmentation phase. This architecture is followed by two parts, each contains two convolutional layers and four deconvolutional layers to predict masks for both streak and globule features.	ISBI 2016 challenge dataset The dataset contains a representative mix of images of both MM and BN – Lesion segmentation: Train_Images (*n* = 900) Test_Images (*n* = 379) – Lesion dermoscopic feature segmentation: Train_Images (*n* = 807) Test_Images (*n* = 335)
Yu et al. (20)	Classification (ALM and BN)	Group A – CNN Sen: 92.57% Spe: 75.39% Acc: 83.51% PPV: 77.14% NPV: 91.88% – Expert Sen: 94.88% Spe: 68.72% Acc.: 81.08% PPV: 73.13% NPV: 93.71%	– Training Data augmentation : 12 patches cropping, rotation, and flipping CNN: 5-layer convolution network + FC – Testing Cropping 12 patches per test image and when one or more images were predicted as containing melanoma, the corresponding test image was interpreted as containing melanoma	Dermoscopic images ALM (*n* = 350) and BN (*n* = 374) – Group A ALM (*n* = 175) and BN (*n* = 187)
Nasr-Esfahani et al. (21)	Classification (melanoma and nevus)	Sen: 81% Spe: 80% Acc: 81% PPV: 75% NPV: 86%	Pre-processing : Removal of noise and illumination artifacts CNN: 2-layer convolution network (20 feature maps and 50 feature maps) + FC	Clinical images Melanoma (*n* = 70) and nevus (*n* = 100) Train_Images (80%) Test_Images (20%)
Premaladha et al. (22)	Classification (MM and BN) Best model : DLNN	Sen. 94.83% Spe. 90.46% Acc. 92.89%	Pre-processing : CLAHE and median filter Segmentation : Normalized Otsu's segmentation (NOS) Classifier : Compared DLNN and hybrid Adaboost-SVM. Best model was DLNN	Dermoscopy images Train_Images (85%) Test_Images (15%)
Matsunaga et al. (23)	Classification (melanoma, nevus and SK)	– ISBI 2017 challenge dataset AUC : 0.958 – ISBI 2016 challenge dataset AUC : 0.874	Pre-processing : luminance and color balance of input images are normalized exploiting color constancy CNN : Fine-tuned 50-layer ResNet MM classifier and SK classifier Ensemble classifier made by merging two classifiers	ISBI 2017 challenge dataset Train_Images : provided data (374 MM, 254 SK, 1372 nevi) + external data (409 MM, 66 SK, 969 nevi) Test_Images (*n* = 150)
Tschandl et al. (24)	Classification (melanoma, BCC, dermatofibroma, melanocytic naevi, seborrheic keratoses and vascular lesion)	– CNN Sen: 90% Spe: 71% AUC: 91% – StudentsSen: 86% Spe: 79% AUC: 85%	All images from the students' training session were also used to retrain the last layer of the “GoogLeNet Inception v3” neural network, without any kind of test-set augmentation (4,000 epochs, learning rate 0.001, batch size 50).	Dermoscopic images (*n* = 348) Train_Images (*n* = 298): melanoma (*n* = 62), BCC (*n* = 40), dermatofibroma (*n* = 7), melanocytic naevi (*n* = 129), SK (*n* = 38), and vascular lesion (*n* = 22) Test_Images (30%): melanoma (*n* = 10), BCC (*n* = 10), dermatofibroma (*n* = 2), melanocytic naevi (*n* = 14), SK (*n* = 9) and vascular lesion (*n* = 5)
Esteva et al. (25)	Classification (757 diseases)	– 3-way classification Dermatologist 1 Acc: 65.6% Dermatologist 2 Acc: 66.0% CNN Acc: 69.4 ± 0.8% CNN – partitioning algorithm Acc: 72.1 ± 0.9% – 9-way classification Dermatologist 1 Acc: 53.3% Dermatologist 2 Acc: 55.0% CNN Acc: 48.9 ± 1.9% CNN – partitioning algorithm Acc: 55.4 ± 1.7%	– Training Using Google Inception v3 CNN architecture pretrained on the ImageNet dataset (1.28 million images of over 1,000 generic object classes) and fine-tuned on their own dataset of 129,450 skin lesions comprising 2,032 different diseases. The 757 training classes were defined using a novel taxonomy of skin diseases and a partitioning algorithm that maps diseases into training classes. – Testing Author developed an algorithm to partition diseases into fine-grained training classes (for example, amelanotic melanoma and acral lentiginous melanoma). During inference, the CNN outputs a probability distribution over these fine classes. To recover the probabilities for coarser-level classes of interest (for example, melanoma) they summed the probabilities of their descendants	Dermoscopic and conventional images (*n* = 129,450) Train_Images (*n* = 127,463) Test_Images (*n* = 1,942)
Lee et al. (26)	Classification (ALM and BN)	– CNN Sen: 90.2% Spe: 93.8% AUC: 97.6% – Board-certified dermatologists Sen: 87.0% Spe: 71.4% Acc: 79.2%	Data augmentation : four center-overlapping patches CNN : Fine-tuned 50-layer ResNet Made an ensemble model [merging Model 2 (intermediate tumor in BN set) and Model 3 (intermediate tumor in ALM set)]	Dermoscopy images ALM (*n* = 500), BN (*n* = 500) and intermediate tumor (*n* = 72) Train_Images (*n* = 872): ALM (*n* = 400), BN (*n* = 400) and intermediate tumor (*n* = 72) Test_Images (*n* = 200): ALM (*n* = 100), BN (*n* = 100)
Cui et al. (27)	Classification (melanoma and non-melanoma)	– CNN (best model: Inception V3) Acc: 93.70% Sen: 95.30% Spe: 92.10%	CNN: Fine-tuned CNNs (AlexNet, VGG16, VGG19, Inception V3) and compared CNNs (best model was Inception V3)	Dermoscopy images deep learning dataset (*n* = 2,200): melanoma (*n* = 564) and non-melanoma (*n* = 1,636)

**Table 2:** Melanoma skin cancer publications using conventional machine learning method.

**Publication**	**End-point**	**Results**	**Method**	**Dataset**
Sabouri et al. (28)	Classification (MM and BN)	Sen: 89.28% Spe: 100% (best model: cascade classifier)	Pre-processing: Artifact removal (hair artifact removal) Cropping (512*512) and lesion segmentation (border segmentation) Feature extraction: Color features: RGB and HSV Texture features: using GLCM Classifier: compared many models (KNN, MLP, Naïve Bayes, RF, SVM). Best model was cascade SVM Classifier (SVM #1 using normalized HSV, SVM #2 using a combination of color and texture features)	Unspecified images Train_Images (*n* = 370): MM (*n* = 175) and BN (*n* = 195) Test_Images (*n* = 42): MM (*n* = 16) and BN (*n* = 26)
Kaur et al. (29)	Pink lesion classification within MM or BN	AUC: 0.879 (all features)	Relative color thresholds Segment of 3 shades of pink (light, dark and orange pink) Quintile overlays Feature extraction: Blob features (5 per shade) Color features for each pink shade over entire lesion (15 per shade) Texture features derived from lesion histogram (24 per shade) Location features (6 per shade) Classifier : multivariate analysis using linear regression was performed using the Proc Logistic function in SAS	Dermoscopic images. Train_Images (*n* = 60): Only MM containing visible pink areas within the lesion Test_Images (*n* = 132): MM (*n* = 54), benign dysplastic, and congenital nevi (*n* = 78)
Shimizu et al. (30)	4-class classification (melanoma, nevus, BCC, SK) Best model: Layered model	Acc: 0.904 AUC: 0.864 (AUC denotes the area of the receiver-operating characteristic (ROC) curve between %M and min (%N, %B, %S).)	Border detection : The core of the algorithm was color thresholding, removal of artifacts such as microscope border and hair, and inclusion of bright area seen specifically in NoMSLs (BCC and SK) Feature extraction: Color-related Features : calculating ten statistics for the intensity of six color channels (RGB, HSV) Subregion-related features : describing geometrical distribution of the color. Texture-related features : by adopting GLCM Classifier: compared layered model and flat model.	Dermoscopic images Train_Images (not described) Test_Images (*n* = 964): melanoma (*n* = 105), nevi (*n* = 692), and SK (*n* = 98), BCC (*n* = 69)
Abedini et al. (31)	Classification (MM and BN)	Acc: 0.90 (accuracy continues to improve with some fluctuation before converging at approximately 90% after 150 responses.)	One feature of the system enables the domain expert to improve previously built models. Classifier with a stochastic gradient descent SVM and a feedback mechanism. Eventually, as more feedback is provided (more training examples), the SVM accuracy improves.	Dermoscopic images Train_Image (*n* = 100) Test_Image (*n* = 5): melanoma (*n* = 3) and normal skin (*n* = 2)
Marchetti et al. (32)	Classification (MM and BN) Best model: greedy fusion	– Greedy fusion Sen: 58% Spe: 92% AUC: 86% – Average dermatologist Sen: 82% Spe: 59% AUC: 71%	Compared five methods of fusing all automated predictions from the 25 participating teams in the ISBI challenge into a single prediction (three machine learning methods and two non-learned approaches)	ISBI 2016 challenge dataset The dataset contains a representative mix of images of both MM (*n* = 248) and BN (*n* = 1,031) Train_Images (*n* = 900) Test_Images (*n* = 379) Reader study images (*n* = 100): MM (*n* = 50) and BN (*n* = 50)

**Table 3:** Melanoma skin cancer publications using hybrid method.

**Publication**	**End-point**	**Results**	**Method**	**Dataset**
Jafari et al. (33)	Lesion segmentation	Sen: 95.2% Spe: 99% Acc: 98.7%	– Training Pre-processing: edge-preserving smoothing Patch selection: Lesion patch selection Border patch selection Normal skin patch selection CNN: local texture analysis + general structure analysis – Testing Pre-processing: edge-preserving smoothing Patch selection: Global and local patch definition CNN: local texture analysis + general structure analysis Post-processing : selecting largest connected component, dilation and hole filling	Clinical images (*n* = 126) MM (*n* = 66) and non-MN (*n* = 60) Train_Images (75%) Test_Images (25%)
Xie et al. (34)	Classification (melanoma and nevus)	– Xanthous race dataset Sen: 95% Spe: 93.75% Acc: 94.17% – Caucasian race dataset Sen: 83.33% Spe: 95% Acc: 91.11%	Pre-processing : hair removal (using partial differential equation) Segmentation: using SGNN Feature extraction: Region division on dermoscopy images Description of color, texture and border features Feature normalization and dimensionality reduction Classifier: meta-ensemble model of multiple neural network ensembles Ensemble 1: single-hidden-layer BP nets with same structures Ensemble 2: single-hidden-layer BP nets and fuzzy nets Ensemble 3: double-hidden-layer BP nets with different structures	Dermoscopy images – Xanthous race dataset (*n* = 240): MM (*n* = 80) and BN (*n* = 160) – Caucasian race dataset (n = 360): MM (*n* = 120) and BN (*n* = 240)
Sabbaghi et al. (35)	Classification (MM and BN)	Sen: 95.5% Spe: 94.9% Acc: 95% (Deep auto-encoder with BoF)	Each RGB dermoscopy image from a training set is converted to BoF mode Then, the generated BoF (scale-invariant feature transform (SIFT) + color) are fed into the stack auto-encoder for training	Dermoscopic images. MM (*n* = 174) and BN (*n* = 640) Train_Images (*n* = 570) Test_Images (*n* = 244)

#### Deep Learning

Among the deep learning algorithms discussed in the literature, five were fine-tuned using pre-trained models. The remainder were fully trained with new models. In four publications, preprocessing was performed prior to model training. In addition, two publications performed lesion segmentation and classification or segmentation of dermoscopic features. To measure the model performance, one publication (Tschandl, Kittler et al.) compared the results of final-year medical students with those of the model; two publications (Yang et al. and Lee et al.) used the results of dermatological experts as the comparison. From these, Lee et al. showed that experienced dermatologists and inexperienced dermatologists improved their decision making with the help of deep learning models. One publication (Premaladha and Ravichandran) compared the conventional machine learning method 'Hybrid Adaboost-SVM' and a deep learning-based neural network on the same dataset; they showed that the deep learning-based neural network delivered superior performance. Moreover, one publication (Cui et al.) demonstrated that when more data was used, the results of deep learning outperformed conventional machine learning methods.

#### Conventional Machine Learning

From the conventional machine learning publications, four of the five publications performed feature extraction and then created a classifier. Two of these publications used SVM for the classifier, one used multivariable linear regression, and one used a layered model. In three publications, artifact removal or lesion segmentation were performed prior to feature extraction. On the other hand, one publication (Marchetti, Codella et al.) presented a new model using a fusion method, developed by 25 teams participating in International Symposium on Biomedical Imaging (ISBI) 2016.

#### Hybrid (Deep Learning + Machine Learning)

In the publications using hybrid methods, one publication (Jafari, Nasr-Esfahani et al.) preprocessed the input images, extracted the patches, and performed segmentation using a convolutional neural network (CNN). In one publication (Xie, Fan et al.), segmentation was performed after preprocessing, using a neural network called self-generating neural network (SGNN); they then presented an ensemble network by designing a feature extractor and classifier. Furthermore, in one publication (Sabbaghi et al.), a deep auto-encoder combined with bag of features (BoF) outperformed the model using a BoF or deep auto-encoder alone.

### Non-melanoma Skin Cancer: BCC, Squamous Cell Carcinoma (SCC)

We identified seven deep learning publications, three machine learning publications and three hybrid publications on non-melanoma skin cancer. Several publications discussed MM; however, all of them discussed BCC and three publications discussed SCC, thus we classified the publications into these categories. The results are organized in Tables 4–6.

**Table 4:** Non-melanoma skin cancer publications using deep learning method.

**Publication**	**End-point**	**Results**	**Method**	**Dataset**
Rezvantalab et al. (36)	Compare ability of deep learning with the performance of highly trained dermatologists	– Melanoma AUC 82.26% (Dermatologist) 93.80% (DenseNet 201) 94.40% (ResNet 152) 93.40% (Inception v3) 93.20% (Inception ResNet v2) – Basal cell carcinoma AUC 88.82% (Dermatologist) 99.30% (DenseNet 201) 99.10% (ResNet 152) 98.60% (Inception v3) 98.60% (Inception ResNet v2)	Pre-trained Inception v3, Inception ResNet v2, ResNet 152, DenseNet 201	*n* = 10,015 dermoscopic images. Melanoma (1,113 samples) Melanocytic nevi (6,705 samples) BCC (514 samples) Actinic keratosis and intraepithelial carcinoma (327 samples) Benign keratoses (1,099 samples) Dermatofibroma (115 samples) Vascular lesions (142 samples) n_train = 70% n_val, n_test = 15%
Zhang et al. (37)	Automatically classify dermoscopic images for clinical decision support	– Dataset A Acc: 86.54% – Dataset B Acc: 85.86%	GoogLeNet Inception v3 Pre-trained on over 1.28 million images and adjusted the final layer to input own datasets using transfer learning	*n* = 1,067 dermoscopic images Dataset A 418 (Nevus) 291 (SK) 132 (BCC) 226 (Psoriasis) Dataset B 132 (Nevus, SK, BCC, Psoriasis) n_train = 80% n_val, n_test = 10%
Vander Putten et al. (38)	Demonstrate a sensitivity and specificity that could make neural networks a realistic tool for dermatologists	Classification layer 53 layers AUC 0.92, Sen 0.98, Spe 0.95 98 layers AUC 0.89, Sen 0.98, Spe 0.94 152 layers AUC 0.93, Sen 0.97, Spe 0.96	1. Segmentation (deep residual network) 2. Classification (very deep residual network)	Two independent sources (BCC) Dermoscopic images1. “Skin Lesion Analysis Toward Melanoma Detection” competition released with ISBI 2016 2. International Skin Imaging Collaboration (ISIC) Archive
Mandache et al. (39)	Propose exploiting FFOCT images	AUC: 95.93% Sen: 95.2% Spe: 96.54%	**Feature extractor** – Convolutional blocks – Dropout layer – ReLU **Classifier** – Fully connected layer – Dropout layer	*n* = 40 FFOCT images 10 (BCC)
Zhang et al. (40)	Machine learning algorithms need to be combined with sufficient clinical expertise in order to achieve an optimal result	– Dataset A Acc: 87.25% – Dataset B Acc: 86.63%	Developed algorithm based on pre-trained GoogLeNet Inception v3 In order to facilitate decision-making and improve the accuracy algorithm, this summarized classification/diagnosis scenarios based on domain expert knowledge and semantically represented them in a hierarchical structure	*n* = 1,067 dermoscopic images Dataset A 418 (Nevus) 291 (SK) 132 (BCC) 226 (Psoriasis) Dataset B 132 (Nevus, SK, BCC, Psoriasis) n_train = 80% n_val, n_test = 10%
Yap et al. (41)	A method which combines multiple imaging modalities together with patient metadata	– Melanoma AUC: 86.1% – Cancer AUC: 88.8%	Used pre-trained modified ResNet-50 architecture (to extract image features) Using a late fusion technique Image feature vectors were concatenated together with the metadata feature vectors and sent through the embedding network	*n* = 2,917 (metadata + macroscopic images + dermoscopic images) 1,127 (Nevus) 727 (All cutaneous melanomas except mucosal and ocular) 647 (BCC) 273 (SCC) 143 (BKL)

**Table 5:** Non-melanoma skin cancer publications using conventional machine learning method.

**Publication**	**End-point**	**Results**	**Method**	**Dataset**
Marvdashti et al. (42)	Fully automated procedure to detect BCC in *ex-vivo* human skin from PS-OCT images	AUC: 97.2% Sen: 95.4% Spe: 95.4%	Extracting image features from the two complementary image contrasts offered by PS-OCT, intensity and phase retardation (PR) using machine learning Then, classify image features using SVM with linear and Gaussian kernels, KNN, and RF	*n* = 520 PS-OCT 260 (Healthy, 26 patients) 260 (BCC, 26 patients)
Kharazmi et al. (43)	Detection and segmentation of cutaneous vasculature from dermoscopy images and extracted vascular features are explored for skin cancer classification	– BCC AUC: 96.5% – Non-BCC AUC: 96.5%	Segment vascular structures by decomposing the image using ICA, k-means clustering Then, a vessel mask is generated as a result of global thresholding Vascular features fed into an RF classifier (decision tree)	*n* = 659 dermoscopy images 299 (BCC) 360 (Non-BCC)
Kefel et al. (44)	Automatic method for detection of pink blush (common feature in BCC) Manually created borders vs. automatic created borders	Manual AUC: 87.8% Automatic AUC: 87.7%	Border detection by GAC and modified Otsu's threshold Classification: logistic regression by Proc Logit of SAS (smoothness, brightness)	*n* = 2,266 dermoscopic images manually created borders n_train = 354 n_test = 1,024 GAC n_train = 888 n_test = 1,024

**Table 6:** Non-melanoma skin cancer publications using Hybrid method.

**Publication**	**End-point**	**Results**						**Method**	**Dataset**
Annan et al. (45)	Propose BCC detection method	Proposed method AUC (Best model : VGG-16)						1. Graph based skin surface segmentation 2. Surface flattening 3. Deep feature extraction (pre-trained AlexNet, GoogLeNet, VGG-16, VGG-19) BCC classification (PCA, SVM)	*n* = 5,040 OCT images 1,875 (lesion or irregular structure)
		**ConvNet**	**Ori**	**Dimension of PCA feature**					
				**100**	**200**	**500**	**1,000**		
		AlexNet	0.916	0.897	0.915	0.897	0.917		
		GoogLeNet	0.744	0.744	0.744	0.744	0.744		
		VGG-16	0.935	0.858	0.913	0.928	0.931		
		VGG-19	0.891	0.798	0.824	0.863	0.894		
Sarkar et al. (46)	Novel state of the art deep neural network for skin carcinoma detection	– BCC AUC: 97.9% Spe: 97.5% Sen: 98.3% Precision: 96.7% F1 score: 97.5% – Benign AUC: 97.9% Spe: 98.6% Sen: 96.6% Precision: 98.3% F1 score: 97.5%						Pre-processing: Denoising of images by Gaussian blurring Enhancement of images by CLAHE algorithm and use parallel deep residual network (RMSprop optimizer) for classification	n = 700 dermoscopic images 300 (BCC positive) 100 (augmented set of SCC positive) 300 (benign skin lesion) n_train = 560 n_val = 140
Dorj et al. (47)	Focus on the task of the classifying skin cancer	**Cancer**			**AUC, %**	**Sen, %**	**Spe, %**	Pre-trained AlexNet is used to extract training features and the obtained convolutional neural network features are classified into four groups using error-correcting output codes (ECOC), SVM	*n* = 3,753 collected from the internet Actinic Keratoses – 712 (Train), 185 (Test) BCC – 728 (Train), 193 (Test) SCC – 777 (Train), 200 (Test) Melanoma 768 (Train), 190 (Test)
		Actinic Keratoses			92.3	98.9	91.67		
		Basal cell carcinoma			91.8	97.7	86.73		
		Squamous cell carcinoma			95.1	96.9	94.17		
		Melanoma			94.2	97.83	90.74		

The results of all publications were presented using an accuracy indicator, and some of these publications using a variety of indicators, such as specificity, sensitivity, precision, and F1 score. The datasets used in each publication were different, making it impossible to compare them directly.

#### Deep Learning

Rezvantalab et al. compared the abilities of deep learning against the performances of highly trained dermatologists. This publication presented outcomes from various deep learning models. In the BCC classification, the highest AUC of the publication was reported as 99.3%, using DenseNet 201. When compared against dermatologists (AUC 88.82%), the results of deep learning were found superior.

Five publications used datasets of dermoscopic images. One used full-field optical coherence tomography (FFOCT) images, and Jordan Yap et al. used different forms of data including metadata, macroscopic images, and dermoscopic images. Next, they trained a deep learning model using fusion techniques, in which image feature vectors were concatenated with the metadata feature vectors. Two publications by Zhang et al. written in 2017 and 2018, showed interesting results; the 2018 publication improved the previous year's algorithm for utilizing medical information. Their results showed an average improvement of 0.7% over those of the previous year.

#### Conventional Machine Learning

We identified four publications that used only machine learning techniques. Three publications used dermoscopic images and one used polarization-sensitive optical coherence tomography (PS-OCT) images. Each author used different methods and features.

Marvdashti et al. performed feature extraction and classification using multiple machine learning methods [SVM, k-nearest neighbor (KNN)]. Kharazmi et al. segmented vascular structures using independent component analysis (ICA) and k-means clustering, then classified them using a random forest classifier. Kefel et al. introduced automatically generated borders using geodesic active contour (GAC) and Otsu's threshold for the detection of pink blush features, known as a common feature of BCCs. Subsequently, they classified using logistic regression, based on features such as smoothness and brightness.

#### Hybrid (Deep Learning + Machine Learning)

Three publications implementing hybrids were identified. Each publication used a different dataset. One publication used optical coherence tomography (OCT) images and another used dermoscopic images. Unusually, the third publication used data downloaded from the Internet, not directly taken.

Annan Li et al. used deep learning for feature extraction, then classified images using the principal component analysis (PCA) and SVM machine learning techniques. They compared deep learning models and assessed the differences in dimensions of the PCA features. Sarkar et al. applied Gaussian blurring to denoise the images and then used the contrast-limited adaptive histogram equalization (CLAHE) algorithm to enhance them. Unlike previous publications, deep learning was used for classification.

## Implementation in Smartphones

With the spread of smartphones, the mobile application market has expanded rapidly. Applications can be used in various fields, particularly in the field of dermatology through the use of smartphone cameras. In particular, due to the ubiquity of smartphones, easily accessible mobile apps can make it more efficient to detect and monitor skin cancers during the early stages of development. In addition, with the recent development of smartphone processors and cameras, machine learning techniques can be applied, and skin cancer diagnoses can be conducted through smartphones. Table 7 shows that a lot of research and development on smartphone implementation is carried out. AI technology relevant to skin cancer diagnosis is anticipated to eventually be implemented in smartphones, enabling the reduction of unnecessary hospital visits. Many types of mobile health application are already available.

**Table 7:** Smartphone applications.

**Application name**	**Algorithm**	**Evidence**	**Performance**		**References**
DermaCompare (removed)	Machine learning	Not found	Not found		[1]
Lubax (removed)	Content-based image retrieval (compare), KNN (classification)	One peer-reviewed supporting publication	Sensitivity (95% CI) 90% (86–94)	Specificity (95% CI) 92% (85–95)	(48) [2]
MskinDoctor (removed)	Grab cut algorithm (segmentation), SVM (classification)	Not found	Not found		[3]
MySkinMap (removed)	Machine learning	Not found	Not found		[4]
SkinScan	Image processing technique, ABCDE rule	Not found	Not found		[5]
SkinVision	Conditional generative adversarial neural network (segmentation) and SVM (classification)	Two peer-reviewed supporting publications, evaluated in independent publications	Sensitivity (95% CI) iOS: 50% (22–78) Android: 72%(58–87)	Specificity (95% CI) iOS: 50% (22–78) Android: 27% (1–56)	(49–52) [6]
SpotMole	Image processing techniques, ABCDE rule	Evaluated in independent publications	Sensitivity (95% CI) 43% (28–58)	Specificity (95% CI) 80% (60–100)	(52) [7]

*[1]AppAdvice. Derma Compare by Emerald Medical Applications. Available online at: https://appadvice.com/app/derma-compare/982517772*.

*[2]AppAdvice. Lubax - Skin Lesion ID Using Image Recognition by Lubax, Inc. Available online at: https://appadvice.com/app/lubax-skin-lesion-id-using-image-recognition/956423382*.

*[3]AppBrain. mSkin Doctor Mobile Application for Skin Cancer Detection by Aleem Technologies. Available online at: https://www.appbrain.com/app/mskin-doctor/com.maleemtaufiq.mSkinDoctor*.

*[4]AppAdvice. MySkinMap by Xyrupt Technologies, LLC. Available online at: https://appadvice.com/app/myskinmap/1151655127*.

*[5]AppAdvice. SkinScan by TeleSkin ApS. Available online at: https://appadvice.com/app/skinscan/1025190936*.

*[6]SkinVision. Available online at: https://www.skinvision.com/*.

*[7]Google Play. SpotMole. Available online at: https://play.google.com/store/apps/details?id=com.spotmole&hl=nl*.

### Types and Accuracies of Diagnostic Applications Using a Smartphone

According to a recent review (53, 54), numerous applications have already been released, seven of which use image analysis algorithms. Four of the seven applications are not supported by scientific evidence, and these four have been deleted from the app store since the review was conducted; the other three apps are still available. Table 7 provides a summary of the apps. SkinScan, SkinVision, and SpotMole are currently available. SkinVision uses machine learning algorithms and SkinScan and SpotMole use the ABCDE rule (that is asymmetry, border irregularity, color that is not uniform, diameter >6 mm, and evolving size, shape or color). Only one application employs a machine learning technique. The sensitivity and specificity of these applications are shown in the table.

Most diagnosis applications are not accurate (55). Furthermore, only a few inform users using image analysis and machine learning. Most apps are not supported by scientific evidence and require further research.

### Problems and Possible Solutions

Inaccuracies in medical applications can result in problems of legal liability. In addition, the transmission of patient information may correspond to telemedicine practices, for which there are certain legal restrictions; these include information protection regulations to prevent third parties accessing data during the transmission process. Even if the accuracy is improved, the advertisements embedded in the application suggest that the technology could be used for commercial advertisements; for example, to attract patients.

To solve this problem, a supervisory institution in which doctors participate is required, along with a connection to remote medical care services. The United States has been steadily attempting to promote telemedicine in its early stages, to address the issue of access to healthcare. Since the establishment of the American Telemedicine Association (ATA)—a telemedicine research institute—in 1993, legislation, including the Federal Telemedicine Act, has been established. It has been applied to more than 50 detailed medical subjects, including heart diseases, and has been successfully implemented in rural areas, prisons, homes, and schools (56).

To obtain good results, it is necessary to focus on securing high-quality data, to form a consensus between the patient and the doctor, and to actively participate in development.

In summary, the evidence for the diagnostic accuracy of smartphone applications is still lacking because few mHealth apps offer services. In addition, because the rate of service or algorithm change is faster than the peer-review publishing process, it is difficult to compare different apps accurately.

### Risks of Smartphone Applications

Smartphone applications pose some risk to users, especially if the algorithm returns negative results and delays the detection and treatment of undiagnosed skin cancer. It is very difficult to study false-negative rates because there is no histological evidence. Users may not be able to assess all skin lesions, especially if they are located in areas difficult to reach or to see. Given the generally low specificity of current applications, there would be a few false positives. This would put unnecessary stress on the user and result in unnecessary visits to the dermatologist. Furthermore, through limited trust and awareness, the user may not follow the advice provided by the smartphone application.

Chao et al. described the ethical and privacy issues of smartphone applications (57). Whilst applications have the potential to improve the provisions of medical services, there are important ethical concerns regarding patient confidentiality, informed consent, transparency in data ownership, and protection of data privacy. Many apps require users to agree to their data policies; however, the methods in which patient data are externally mined, used, and shared are often not transparent. Therefore, if a patient's data are stored on a cloud server or released to a third party for data analysis, assessing liability in the event of a breach of personal information is a challenge. In addition, it is unclear how the responsibilities for medical malpractice will be determined if the patient is injured as a result of inaccurate information.

## Conclusion

In this review, we analyzed a total of 35 publications. Studies on skin lesions were divided into those assessing malignant melanomas and non-melanoma skin cancers. In addition, studies involving clinical data and OCT images were used alongside those involving the dermoscopic images widely used in dermatology. Because the considered datasets differed between the publications, it was impossible to determine how best to perform the analysis. However, as seen in the publication by Cui et al. deep learning methods obtain better results than conventional machine learning methods if the dataset is large. Also, certain publications have reported comparable or superior results to dermatologist. In particular, recent publications have reported that dermatologists have improved diagnostic accuracy with the help of deep learning (26, 58). Therefore, in the future, computer-aided diagnostics in dermatology will show greater reliance on deep learning methods.

For the convenience of users, the use of a smartphone is necessary. However, an accuracy limitation occurs when applied to smartphones. This problem is due to the limitations of hardware, which used conventional machine learning techniques such as SVM rather than deep learning. However, MobileNet has recently made it possible to use deep learning methods in IoT devices, including smartphones (59). This enables deep learning to be applied to IoT devices for faster performances than large networks, which will lead to more active research into skin lesion detection using applications.

Application inaccuracies can lead to legal problems. To solve this problem, doctors and patients must participate together in the development stage, and an institution for managing and supervising this process is also required.

## Author Contributions

BO and SY: contributed conception and design of the study, wrote sections of the manuscript, and contributed to manuscript revision. YC and HA: collected data and wrote the first draft of the manuscript. All authors read and approved the submitted version.

## Conflict of Interest

The authors declare that the research was conducted in the absence of any commercial or financial relationships that could be construed as a potential conflict of interest.

## Footnotes

^1^http://kosis.kr/statHtml/statHtml.do?orgId=117&tblId=DT_117N_A00025&conn_path=I2

## Abbreviations

ACC: accuracy
AI: artificial intelligence
ALM: acral lentiginous melanoma
AUC: area under the curve
BCC: basal cell carcinoma
BN: benign nevus
BoF: bag of features
CLAHE: contrast-limited adaptive histogram equalization
CNN: convolutional neural network
DCNN: deep convolution neural network
DI: dice score
DLNN: deep learning-based neural network
FFOCT: full-field optical coherence tomography
GAC: geodesic active contour
GLCM: gray-level co-occurrence matrix
ICA: independent component analysis
ILSVRC: ImageNet large-scale visual recognition challenge
ISBI: International Symposium on Biomedical Imaging
ISIC: International Skin Imaging Collaboration
JA: Jaccard index
KNN: k-nearest neighbor
LFN: lesion feature network
LICU: lesion index calculation unit
LIN: lesion indexing network
MLP: multi-layer perceptron
MM: malignant melanoma
NPV: negative predictive values
OCT: optical coherence tomography
PCA: principal component analysis
PPV: positive predictive values
PS-OCT: polarization-sensitive optical coherence tomography
RF: random forest
SCC: squamous cell carcinoma
SGNN: self-generating neural network
SK: seborrheic keratosis
Sen: sensitivity
Spe: specificity
SVM: support vector machine.
